# The Sedative Effect of Propranolol on Critically Ill Patients: A Case Series

**DOI:** 10.3389/fmed.2017.00044

**Published:** 2017-05-04

**Authors:** Junji Shiotsuka, Andrew Steel, James Downar

**Affiliations:** ^1^Division of Critical Care, University Health Network, Toronto, ON, Canada; ^2^Department of Anaesthesiology, University Health Network, Toronto, ON, Canada

**Keywords:** propranolol, adrenergic beta-antagonists, delirium, psychomotor agitation, intensive care unit, hypnotics and sedatives

## Abstract

**Introduction:**

Recent studies have examined the effectiveness of alpha-2 adrenergic agonists for controlling delirium and agitation. Propranolol, a non-selective beta-adrenergic antagonist with good penetration of the blood–brain barrier, has not been investigated for this purpose.

**Materials and methods:**

We retrospectively reviewed the medical records of all patients who were prescribed propranolol in our Medical Surgical ICU from January 1, 2010, to December 31, 2013. We recorded the sedation level and daily dose of sedatives, analgesics, and antipsychotics administered each day for 6 days after starting propranolol, and compared them to the day before starting propranolol.

**Results:**

Sixty-four patients met inclusion criteria. Thirty-eight episodes met exclusion criteria, leaving 27 patients (31 episodes). The administration of propranolol was associated with significant reductions in fentanyl equivalents (65%, *p* = 0.009), midazolam equivalents (57%, *p* = 0.048), propofol (16%, *p* = 0.009), and haloperidol (44%, *p* = 0.024) on day 2 after starting propranolol compared with baseline. A stratified analysis showed that these decreases were seen regardless of clinical improvement or deterioration.

**Conclusion:**

The use of propranolol was associated with a significant reduction in doses of sedatives and analgesia. Further studies are needed to determine whether propranolol may be a useful adjuvant for managing delirium and agitation in the ICU.

## Introduction

Hyperactive delirium is a common problem in the ICU setting, particularly among more physiologically stressed patients. Practice guidelines have been published to help clinicians manage agitation and delirium, and these guidelines suggest an approach that includes analgesia, non-benzodiazepine sedatives, and possibly atypical antipsychotics (1). However, these medications can be insufficient to treat some cases of delirium. The pathophysiology of delirium is not fully understood, but several neurotransmitters are known to play an important role, including catecholamines (2, 3). The locus coeruleus (LC) is a small pontine nucleus that provides brain norepinephrine and plays an important role in regulating the sleep–wake cycle and arousal state. The norepinephrine provided by the LC acts at the medial septal area (MSA) and the medial preoptic area (MPOA) to activate waking-active neurons (*via* alpha-1 adrenergic receptors) and inhibit sleep–active neurons (*via* alpha-2 adrenergic receptors). There are also beta-1, beta-2, and beta-3 adrenergic receptors in the MSA and MPOA (4).

Dexmedetomidine is a potent alpha-2 adrenergic agonist that binds to the alpha-2 adrenergic receptor subtype A at the LC, resulting in almost complete inhibition of the LC, which has a sedative effect (5, 6). Many investigators have used dexmedetomidine and clonidine (another alpha-2 agonist) to control agitation and delirium (7–15). Beta-2 adrenergic receptor activation also appears to be important in the MSA and MPOA, resulting in dose-dependent increases in time spent awake (4). Beta-blocking agents have been less well studied for ICU delirium, although beta-blockers are known to have beneficial effects on anxiety, posttraumatic Stress Disorder (PTSD) and aggressive behavior in a variety of populations (16–20). Propranolol is a non-selective beta-adrenergic antagonist that has good penetration of the blood–brain barrier (21). In our institution, propranolol has been used with a sedative intent for cases of refractory agitated delirium or for patients who cannot be weaned from our usual sedative regimen. The purpose of this study was to determine whether propranolol had a sedative effect on these critically ill ICU patients. We hypothesized that propranolol administration would be associated with a reduction in the use of sedatives, analgesics, and antipsychotics.

## Materials and Methods

### Study Design: Retrospective Case Series

#### Patients

All patients who were prescribed propranolol in the Medical Surgical Intensive Care Unit (MSICU) at Toronto General Hospital, Toronto, ON, Canada from January 1, 2010, to December 31, 2013. As this study is not a prospective study, the reason for prescription and the dose of propranolol was at the physicians’ descretion. Propranolol is rarely if ever used in our institution to treat tachycardia or hypertension, but we cannot be certain that propranolol was prescribed to treat hyperactive delirium in all cases. The exclusion criteria are as follows; patients younger than 18 years old, those who were given propranolol for less than 48 h, those who were given propranolol on the first day of the MSICU admission, and those who were discharged from the MSICU within 48 h of starting propranolol. Although propranolol has a fairly short plasma half life (1–6 h), we excluded patients who were given propranolol for less than 48 h in order to restrict ourselves to those who likely achieved a steady plasma level (22). Those who died while receiving propranolol were noted, but they were not included in the analysis of medication dose changes as we could not evaluate the association between propranolol discontinuation and the change of dose of sedatives and analgesics. The primary outcome was the relative change in the dose of sedative, analgesic, and antipsychotic over the course of propranolol administration in the MSICU. This study was approved by the Research Ethics Board at the University Health Network.

#### Data Collection

We collected the following data daily from 1 day before propra-nolol was first administered (day −1) to 6 days after propranolol was first administered (day 6); heart rate, mean arterial blood pressure, systolic blood pressure, the daily dose of sedation and analgesia, the Intensive Care Delirium Screening Checklist (ICDSC; we used the worst measurement in each 24-h period), the Sedation Agitation Score (SAS; we used the worst measurement in each 24-h period), and the Sequential Organ Failure Assessment (SOFA) score. Benzodiazepine and opioid doses were expressed in midazolam and fentanyl equivalence, respectively. The equivalence was calculated as follows: for benzodiazepines, 1 mg midazolam = 0.5 mg lorazepam = 0.25 mg clonazepam = 2 mg diazepam; for opioids, 1µg fentanyl = 0.02 mg i.v. hydromorphone = 0.15 mg oral hydromorphone = 0.1 mg i.v. morphine = 0.58 mg oral morphine = 0.4 mg oral oxycodone (23–29). As this was a retrospective review, the selection of the medications and the order of titration of medications were at the discretion of the treating team. We defined adverse events as follows: initiation or >20% dose increase in vasopressors within 48 h of first propranolol administration, new onset AV block confirmed by ECG, or clinically diagnosed bronchospasm.

We divided patients into three groups according to their response to propranolol. Group 1 (good response) was defined by a dose decrease of at least 50% for at least 1 sedative/opioid/antipsychotics AND all other sedatives/opioids/antipsychotics either increased <50% or decreased within 48 h from the initiation of propranolol. Group 2 (mixed response) was defined by dose changes of <50% for all sedatives/opioids/antipsychotics OR decreases and increases of >50% within 48 h from the initiation of propranolol. Group 3 (poor response) was defined by a dose increase of at least 50% for at least 1 sedative/opioids/antipsychotics AND others were changed less than 50%.

### Statistical Analysis

We used the Wilcoxon Signed Rank test to perform pairwise comparisons of the change in dose equivalence of each sedative/analgesic/antipsychotic between day −1 and each day until day 6, and to compare the change in the ICDSC and the SAS between day −1 and each day until day 6. To look for the effect of clinical improvement on medication dosing, we performed the same comparisons within the subgroups of patients who had improved or worsened SOFA scores in a *post hoc* analysis. We used ANOVA to compare demographics and clinical features of the three groups described above.

## Results

**Figure 1:**
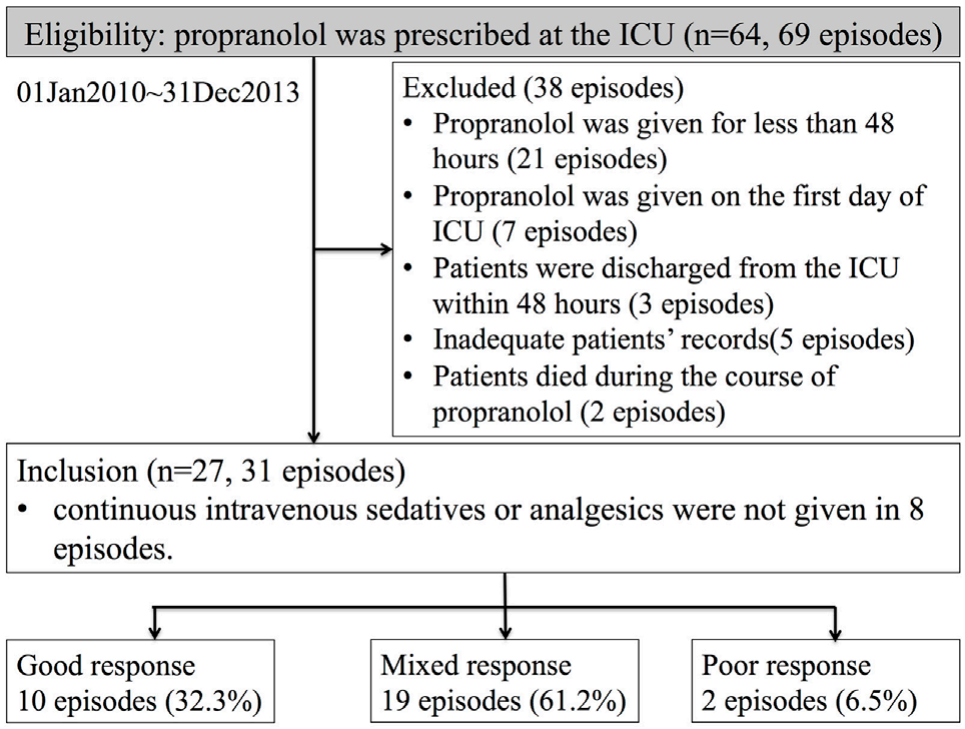
**Numbers of patients who were eligible, excluded, and analyzed**. The definitions of good, mixed, and poor response are explained in the Section “Materials and Methods.”

We identified 64 patients who were prescribed propranolol in the MSICU during the study period (69 episodes). Thirty-eight episodes were excluded by following reasons: propranolol was given for less than 48 h in 21 episodes; propranolol was started on the first day of ICU in 7 episodes; patients were discharged within 48 h from propranolol administration in 3 episodes; patients’ records were inadequate for 5 episodes; patients died during the course of propranolol in 2 episodes. A total of 27 patients (31 episodes) were included in the analysis (Figure 1). Patient characteristics and diagnoses on admission are provided in Table 1. Although there were eight episodes in which patients were not given continuous intravenous sedatives or analgesics, there was no patient who did not receive any sedatives, analgesics, or antipsychotics during the administration of propranolol. Most patients were receiving multiple sedatives, analgesics, and antipsychotics. Benzodiazepines and opioids were coadministered in more than 80% of episodes of propranolol administration (Table 2).

**Table 1:** **Patient demographics**.

Characteristic		
Sex, *n* (%)	Male	16 (59%)
	Female	11 (41%)
	Total	27 (100%)
Age, years, mean (SD)		46.9 (14)
Diagnosis, *n* (%)	Idiopathic pulmonary fibrosis	6 (22%)
	Acute respiratory distress syndrome	9 (33%)
	Pneumonia	5
	Aspiration	1
	Chronic restrictive lung disease	1
	Sepsis	1
	Unknown origin	1
	Idiopathic pulmonary hypertension	3 (11%)
	Others	9 (33%)

**Table 2:** **Analgesics, sedatives, antipsychotics and other psychoactive medications used**.

Medication or class of medication	*N* (%)
Opioids	26 (96)
Benzodiazepines	25 (93)
Quetiapine	20 (74)
Haloperidol	19 (70)
Propofol	16 (59)
Clonidine	12 (44)
Zopiclone	12 (44)
Serotonin-specific reuptake inhibitors	7 (26)
Ketamine	5 (19)
Olanzapine	5 (19)
Diphenhydramine	5 (19)
Dimenhydrinate	4 (15)
Gabapentin	3 (11)
Dexmedetomidine	1 (4)
Bupropion	1 (4)

### Titration of Sedatives, Analgesics, and Antipsychotics

**Figure 2:**
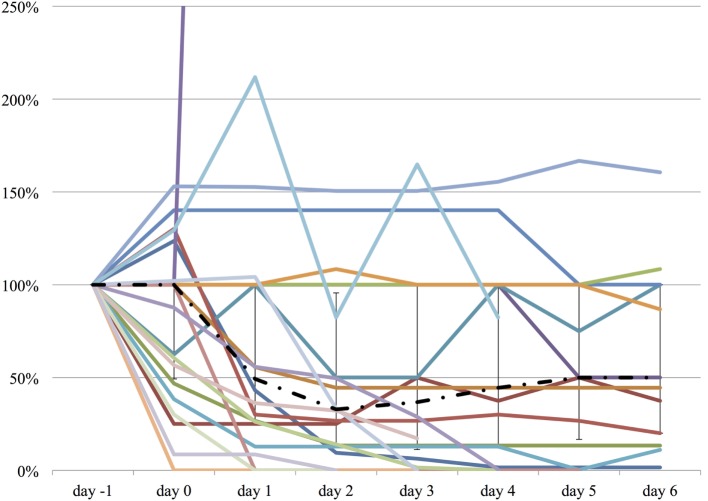
**The relative change in midazolam equivalent dose from day −1 to day 6**. The vertical axis represents percent change of midazolam equivalent of each patient, compared to day −1. The dashed line represents the median midazolam equivalent dose, and the range represents the interquartile range.

**Figure 3:**
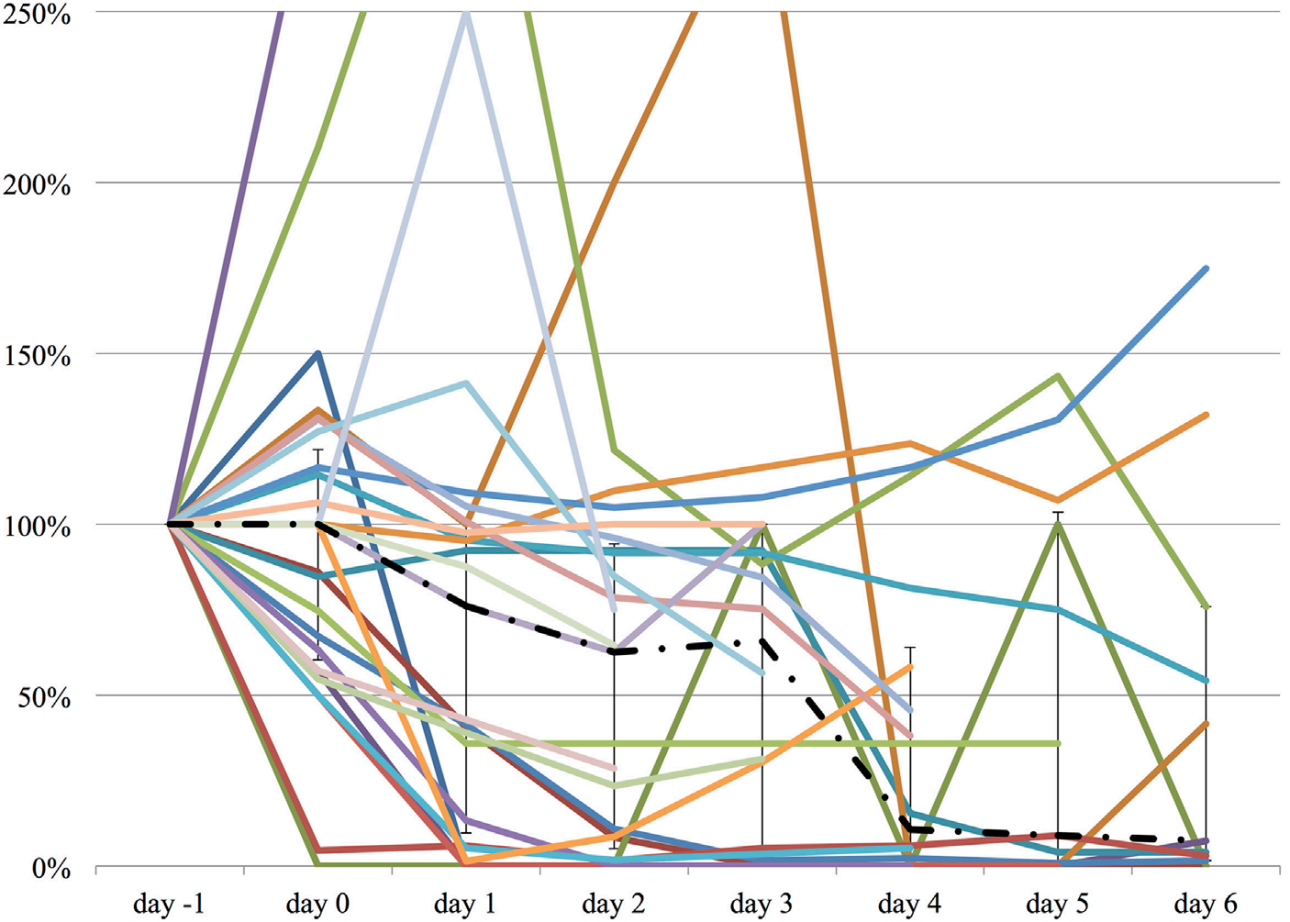
**The relative change in fentanyl equivalent dose from day −1 to day 6**. The vertical axis represents percent change of fentanyl equivalent of each patient, compared to day −1. The dashed line represents the median fentanyl equivalent dose, and the range represents the interquartile range.

**Figure 4:**
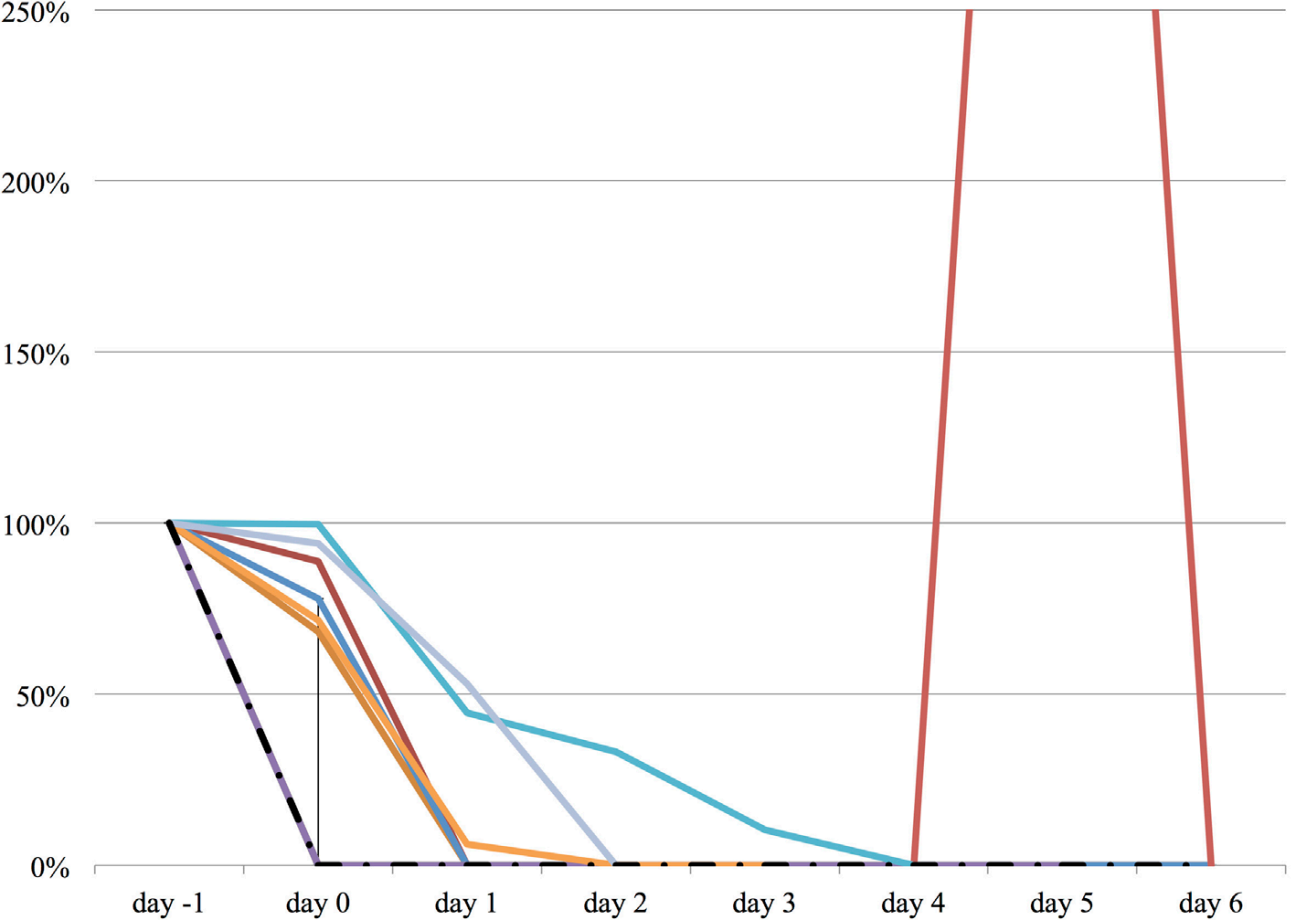
**The relative change in propofol dose from day −1 to day 6**. The vertical axis represents percent change of propofol dose of each patient, compared to day −1. The dashed line represents the median propofol dose, and the range represents the interquartile range.

**Figure 5:**
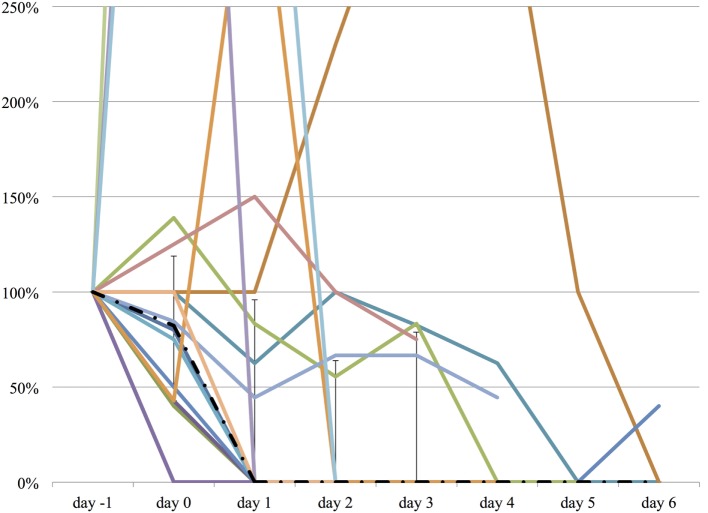
**The relative change in haloperidol dose from day −1 to day 6**. The vertical axis represents percent change of haloperidol dose of each patient, compared to day −1. The dashed line represents the median haloperidol dose, and the range represents the interquartile range.

We measured the difference in doses of five common sedatives/opioids/antipsychotics given between day −1 and each day after propranolol administration until day 6. Mean midazolam equivalence decreased significantly from 79.21 ± 142.76 mg/day on day −1 to 44.34 ± 121.20 mg/day on day 2 (*n* = 25, *p* = 0.048; Figure 2). Mean fentanyl equivalence decreased significantly from 2,535.14 ± 2,852.83 µg/day on day −1 to 1,646.43 ± 2,608.98 µg/day on day 2 (*n* = 29; *p* = 0.009; Figure 3). Mean propofol dose decreased significantly from 942.59 ± 1,629.30 mg/day on day −1 to 154.48 ± 641.05 mg/day on day 2 (*n* = 29; *p* = 0.009; Figure 4). Mean haloperidol dose decreased significantly from 9.91 ± 13.55 mg/day on day −1 to 4.40 ± 10.13 mg/day on day 2 (*n* = 29; *p* = 0.024; Figure 5). Mean quetiapine dose increased significantly from 62.93 ± 117.76 mg/day on day −1 to 129.31 ± 142.06 mg/day on day 2 (*n* = 29; *p* = 0.019).

### The Effect of Clinical Course on Changes in Medication Dose

Table 3 shows the change in mean medication doses according to clinical course (improved or worsened, as determined by changes in SOFA score). The number of patients included in the analysis was variable depending on medication, and comparison data are shown for either day 2 or 3. The mean opioid dose (shown in fentanyl equivalence) decreased significantly in both groups. The mean benzodiazepine dose (shown in midazolam equivalence) also decreased in both groups, although the change was not significant in either. The mean propofol dose decreased in both groups, but the change was only significant in patients who improved clinically. The mean haloperidol dose decreased significantly among the patients who deteriorated, but did not change among the patients who improved. The mean quetiapine dose increased significantly in patients who deteriorated, but did not change in patients who improved.

**Table 3:** **Changes in medication dosing among patients who improved or deteriorated clinically**.

	Sequential organ failure assessment (SOFA) score was same or worse than day −1		SOFA score was better than day −1	
Fentanyl equivalence (µg/day)	Day −1 (*n* = 13)	Day 3 (*n* = 13)	Day −1 (*n* = 12)	Day 2 (*n* = 12)
	1,440 (175–4,800)	100 (25–2,400)*	3,595 (1,950–5,190)	965 (252.5–4,440)*
Haloperidol (mg/day)	Day −1 (*n* = 13)	Day 3 (*n* = 13)	Day −1 (*n* = 14)	Day 3 (*n* = 14)
	10.0 (0–20.0)	0 (0–0)*	2.5 (0–10.0)	0 (0–0)
Midazolam equivalence (mg/day)	Day −1 (*n* = 13)	Day 3 (*n* = 13)	Day −1 (*n* = 14)	Day 3 (*n* = 14)
	17.0 (1.0–128.0)	4.0 (0–16.0)	8.0 (0.5–28.5)	6.0 (0.5–30.4)
Propofol (mg/day)	Day −1 (*n* = 19)	Day 2 (*n* = 19)	Day −1 (*n* = 12)	Day 2 (*n* = 12)
	0 (0–0)	0 (0–0)	590.0 (33.8–3,237.5)	0 (0–0)*
Quetiapine (mg/day)	Day −1 (*n* = 13)	Day 3 (*n* = 13)	Day −1 (*n* = 14)	Day 3 (*n* = 14)
	0 (0–0)	100.0 (0–200.0)*	0 (0–181.3)	75.0 (0–187.5)

*Data are presented as the median and interquartile range*.

*p < 0.05 for comparison with day −1 (Wilcoxon Signed-Rank test).

### Effect on Delirium and Agitation

**Figure 6:**
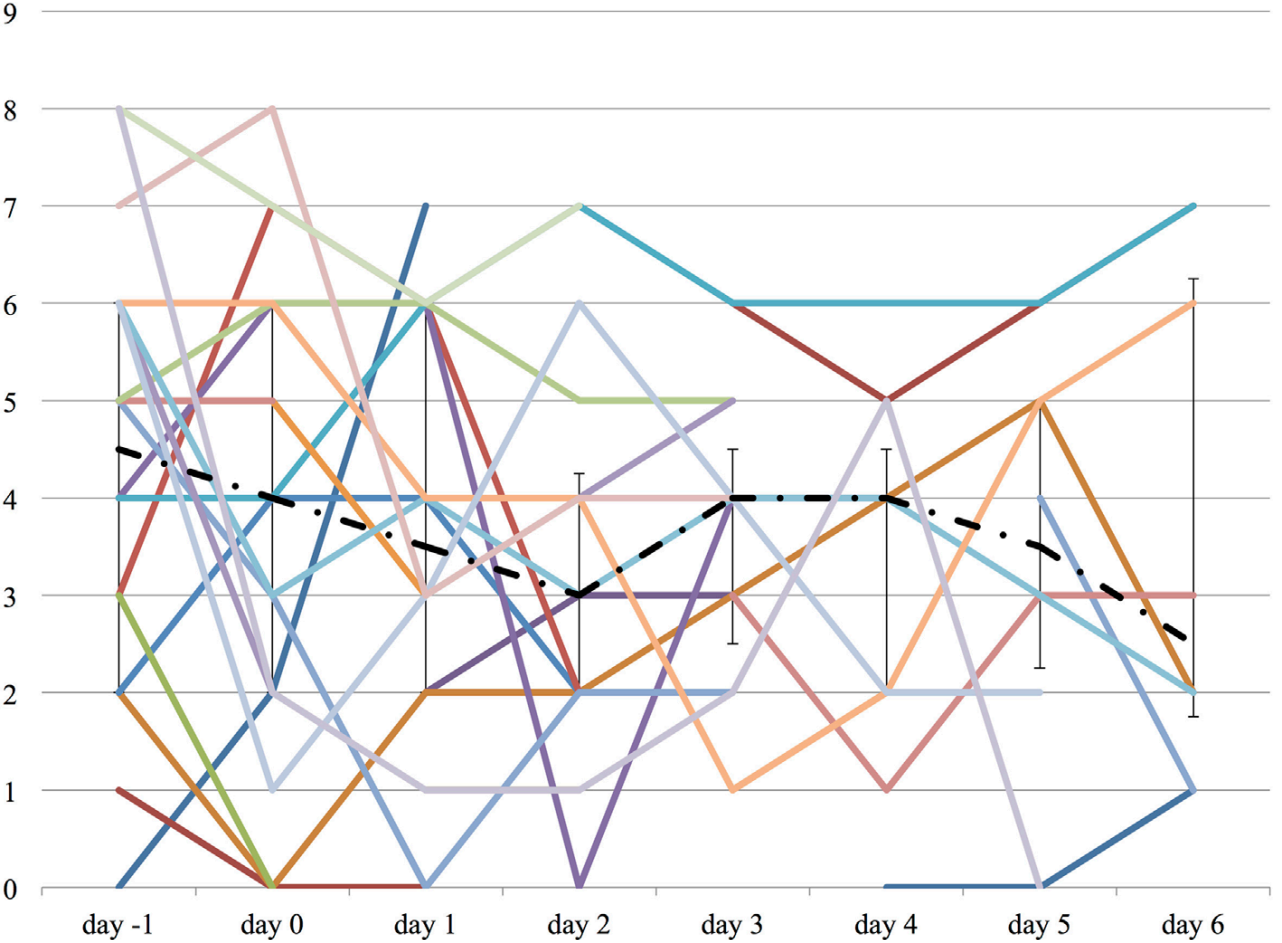
**The worst Intensive Care Delirium Screening Checklist (ICDSC) score on each day from day −1 to day 6**. The vertical axis represents ICDSC score of each patient. The dashed line represents the median ICDSC score, and the range represents the interquartile range.

**Figure 7:**
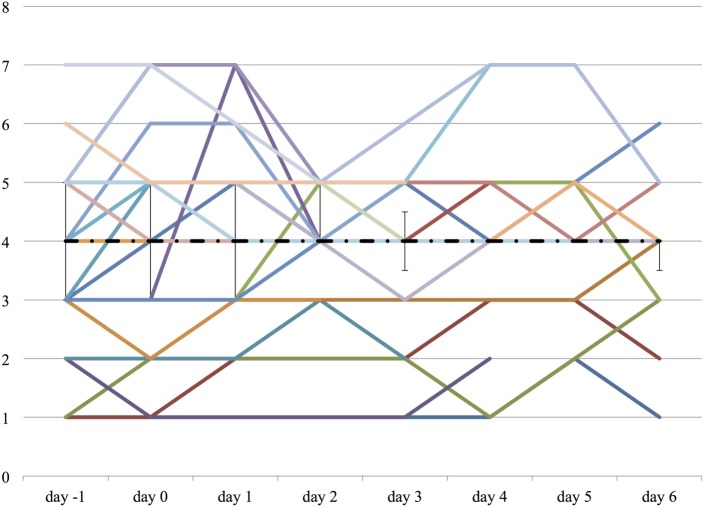
**The worst Sedation Agitation Score (SAS) score on each day from day −1 to day 6**. The vertical axis represents SAS score of each patient. The dashed line represents the median SAS score, and the range represents the interquartile range.

Both the ICDSC and the SAS were not significantly changed over the course of propranolol despite the reduction of the medications described above (Figures 6 and 7).

### Response to Propranolol Administration

According to the criteria described in the methods section, we determined that 10 episodes (32.3%) fell into Group 1 (good response), 19 episodes (61.2%) fell into Group 2 (mixed response), and 2 episodes (6.5%) fell into Group 3 (poor response). There was no significant difference in age, gender, and changes in heart rate between the groups, but patients in Group 1 received significantly higher doses of propranolol than patients in the other groups (Group 1: 103.7 ± 50.3 mg, Group 2: 76.3 ± 38.9 mg, Group 3: 25.0 ± 45.4 mg; *p* = 0.05).

### Adverse Effects

There were five episodes (13%) of prespecified adverse effects. Four episodes involved the initiation or >20% dose increase in vasopressors within 48 h of starting propranolol. One patient had two separate episodes of bradycardia following the initiation of propranolol. On both occasions, the bradycardia resolved when the propranolol was discontinued. There were no recorded episodes of bronchospasm after propranolol administration. Two patients died during the course of propranolol. One patient was admitted to the ICU for ARDS secondary to community acquired pneumonia and suspected lung transplant rejection. The patient had a remote history of double lung transplantation for alpha-1 antitrypsin deficiency. She required mechanical ventilation, became agitated, and required increasing sedation for several days before death. The ICU physician added propranolol for agitation. After 6 h the patient’s hypoxemia began to worsen, and after 24 h the patient suddenly developed bradycardia and pulseless electrical activity and could not be resuscitated. The last dose of propranolol was given 7 h prior to death. The attending physician recorded hypoxic respiratory failure as the cause of death. The second patient was admitted to the ICU for *Pneumocystis jirovecii* pneumonia and cytomegalovirus infection with a remote history of liver transplantation. Six days prior to death, the patient developed febrile neutropenia and tachycardia (120–140 bpm) and was treated empirically for bacterial infection. Propranolol was given apparently to control tachycardia and was well-tolerated for the first 48 h. After 60 h, the patient suddenly became hypotensive and bradycardic, with an ECG revealing tall T waves. Shortly thereafter, the patient developed a cardiac arrest and could not be resuscitated. The last dose of propranolol was 7 h before the death, and the attending physician recorded myocardial infarction as the cause of death.

## Discussion

In the present study, we found reductions in mean doses of sedatives and opioids after the initiation of propranolol in critically ill patients, and that almost one-third of patients who received propranolol had an important reduction in sedative, analgesia, and/or antipsychotic medication doses within 48 h. Notably, the reduction in sedatives and opioids was seen regardless of whether the patient improved or deteriorated clinically over the same period. Adverse events were rare, and although two patients died while receiving propranolol, neither death was apparently related to propranolol. We identified a subgroup of patients whose sedative/analgesic/antipsychotic requirements fell significantly after starting propranolol, but we could not identify any significant demographic or clinical predictors of a good response to propranolol.

Agitation and delirium are serious problems among critically ill patients, but the pathophysiology of delirium is not fully understood. In practice, physicians typically use one or more medications to control delirium and agitation: benzodiazepines and propofol, which act as gamma aminobutyric acid type A (GABA_A_) receptor agonists, antipsychotic medications, such as haloperidol (a dopamine D2 receptor antagonist) or quetiapine (which is a dopamine D2 receptor and serotonin 5-HT receptor antagonist), and opioids, which act mainly as mu opioid receptor agonists (1, 30). Barr et al. recently published clinical practice guidelines for the management of delirium and agitation, based on an exhaustive review of over 19,000 references (1). The pharmaceutical recommendations included an “analgesia-first” sedation approach, using non-benzodiazepines (including dexmedetomidine) as needed to treat agitation. Delirium is thought to be related to excess dopamine activity (2, 3), and the guidelines suggested treatment with atypical antipsychotics.

Previous investigators have used beta-adrenergic blockers to treat central nervous system symptoms. Liu et al. conducted a small randomized controlled trial (*n* = 15) in which they administered either propranolol or placebo 1 h prior to a dental procedure. Patients who received propranolol reported lower pain and anxiety scores on a visual analog scale before, during and after the procedure (16). Vaiva et al. were able to reduce the incidence of PTSD symptoms using propranolol in a small (*n* = 19) non-randomized study of trauma patients (18). Lindgren et al. found that patients with breast cancer or colorectal cancer who were already taking beta-adrenergic blockers had significantly fewer cancer-related intrusive thoughts in an observational study (20). Fleminger et al. published a systematic review of the management of agitation and aggression in patients with acquired brain injury (19). They included four randomized controlled trials of beta-adrenergic blockers, but these were not meta-analyzed due to low study quality and heterogeneity. Of these four small studies (the largest included 21 subjects), two used propranolol and both found it to be effective for controlling agitation and/or aggression. The other two used pindolol, but only one found it to be effective. Dyck and Chung found that propranolol did not have a significant anxiolytic effect compared with diltiazem and placebo in patients undergoing elective surgery, but the use of propranolol was associated with significantly faster cognitive recovery after anesthesia (17). As far as we know, the present study is the first to investigate the effect of propranolol for critically ill patients. Previous studies mentioned above have shown the effects of propranolol on various neurocognitive disorders besides hyperactive delirium, and some of these are associated with delirium in the ICU (16, 19, 20). Therefore, treatment of these conditions may lead to a decrease in the incidence of delirium in the ICU (1, 30).

Delirium has multiple predisposing and precipitating factors, and adrenergic hyperstimulation is only one potential contributor to delirium (1, 3, 30, 31). However, beta-adrenergic blockers may benefit patients in other ways as well. Morelli et al. recently showed a beneficial effect for Esmolol in a randomized controlled trial of patients with septic shock. Patients who received esmolol had better hemodynamic parameters and 28-day mortality than placebo, although it was an open-label, phase 2 trial that did had not set hemodynamic parameters or 28-day mortality as primary outcomes (32). Critically ill patients often have markedly elevated plasma catecholamine levels, and an excess of adrenergic stimulation is associated with organ dysfunction such as stress-related cardiomyopathy (so called Takotsubo cardiomyopathy), pulmonary edema, coagulation disorders, and hyperglycemia (33). If beta-adrenergic blockers can be used to treat these conditions, which are caused by excess catecholamine stimulation during stress (34), they may also be effective for controlling delirium that has catecholamine stimulation as a contributor. Recently, Gardner and Griffiths suggested that propranolol could play a role in treating or preventing PTSD among survivors of ICU admission (35).

The present study has important limitations. First, since it was an observational, retrospective study, there was no standardized titration of sedatives, opioids, and antipsychotics. As a result, the changes in observed medication use may not have been due to a hypnotic effect of propranolol, but rather another effect such as the physiologic response of patients to propranolol (if nurses were using sedatives and analgesics in response to physiologic parameters). Furthermore, a large number of patients were found to have a “mixed response” to propranolol, with some medication doses increasing while others decreased. We are reassured by the fact that the mean dose of almost every medication fell after initiating propranolol, while measures of sedation and delirium did not change. The one notable exception was a significant increase in quetiapine dose among patients who were deteriorating clinically. Although this increase was coupled with a significant decrease in haloperidol dosing, we cannot exclude the possibility that this change (and not the propranolol) was responsible for the sedative/opioid dose reductions seen in this group. The present study is hypothesis generating, and we plan to conduct a prospective study with protocolized titration of sedation, analgesia, and antipsychotic medication in order to control for the effect of these co-interventions. Second, there were no explicit criteria for the use of propranolol, and there was no standard titration protocol for propranolol. Therefore, our study population was heterogeneous, and we cannot exclude the possibility that some patients did not respond to propranolol because it was not given in sufficient dose. Third, this was a retrospective, single-center study with a small number of patients and no control group. As we did not set hyperactive delirium as an inclusion criteria or a primary outcome, we cannot prove or disprove the effectiveness of propra-nolol for treating agitation or delirium in critically ill patients from the present study. However, the present study adds to a growing body of literature suggesting a beneficial effect of anti-adrenergic medications for critically ill patients. As propranolol has a long history of use and is low cost compared to most sedatives, it could represent an important addition to our pharmaceutical options when confronted with refractory delirium or agitation.

## Conclusion

In this hypothesis-generating observational study, we found that the use of propranolol in critically ill patients was associated with significant reductions in the use of sedatives and opioids. We plan to conduct a prospective study with explicit protocols for using propranolol and other sedatives in order to address the limitations of the present study.

## Ethics Statement

This study was carried out without obtaining a written informed consent, because this study is a retrospective cohort study and has a low risk of causing severe harm on the participating patients. There is a risk of violating the patients’ privacy and, therefore, we complied with the guideline of the University Health Network to keep patients’ privacy. The protocol was approved by the Research Ethical Board of University Health Network.

## Author Contributions

JD and AS developed the concept for the study. JD and JS participated in study design. JS performed data acquisition. JS, AS, and JD performed data analysis and drafted and revised the manuscript. All authors approved the final version of the manuscript.

## Conflict of Interest Statement

The authors declare that the research was conducted in the absence of any commercial or financial relationships that could be construed as a potential conflict of interest.
